# Prophylactic efficacy of probiotics on travelers’ diarrhea: an adaptive meta-analysis of randomized controlled trials

**DOI:** 10.4178/epih.e2018043

**Published:** 2018-08-29

**Authors:** Jong-Myon Bae

**Affiliations:** Department of Preventive Medicine, Jeju National University School of Medicine, Jeju, Korea

**Keywords:** Probiotics, Diarrhea, Randomized controlled trials, Meta-analysis

## Abstract

**OBJECTIVES:**

The 2017 guideline for the prevention of travelers’ diarrhea (TD) by the International Society of Travel Medicine suggested that ‘there is insufficient evidence to recommend the use of commercially available prebiotics or probiotics to prevent or treat TD.’ However, a meta-analysis published in 2007 reported significant efficacy of probiotics in the prevention of TD (summary relative risk [sRR], 0.85, 95% confidence interval [CI], 0.79 to 0.91). This study aimed to synthesize the efficacy of probiotics on TD by updating the meta-analysis of double-blind, placebo-controlled, randomized human trials.

**METHODS:**

The search process was conducted by the adaptive meta-analysis method using the ‘cited by’ and ‘similar articles’ options provided by PubMed. The inclusion criteria were double-blind, placebo-controlled, randomized human trials with hypotheses of probiotics as intervention and TD as an outcome. The adaptive meta-analysis was conducted using Stata software using the *csi*, *metan*, *metafunnel*, and *metabias* options.

**RESULTS:**

Eleven articles were selected for the meta-analysis. The sRR was 0.85 (95% CI, 0.79 to 0.91) and showed statistical significance. There was no heterogeneity (I-squared=28.4%) and no publication bias.

**CONCLUSIONS:**

Probiotics showed statistically significant efficacy in the prevention of TD.

## INTRODUCTION

While environmental hygiene has improved more than before, travelers’ diarrhea (TD) remains one of the most important public health issues in the international community as more and more people travel around the world [1]. Although it might be possible to consider administering antibiotics to prevent TD, given that bacteria account for more than 80% of the pathogens that cause TD [2], this causes the problem of antibiotic resistance [3]. According to the guideline published in 2017 by the International Society of Travel Medicine (ISTM) [1], antibiotics are contraindicated for the prevention of TD among the general population.

The guideline also states that there is insufficient evidence to recommend the use of commercially available prebiotics or probiotics to prevent or treat TD. Among 2 meta-analysis studies cited in the guideline [4,5], Sazawal et al. [4] selected 4 articles that published randomized, placebo-controlled trial results [6-9], and revealed that there was no significant difference in prevention efficacy (summary relative risk [sRR], 0.92; 95% confidence interval [CI], 0.80 to 1.06). However, McFarland’s study [5], which was published a year later in 2007, added three articles to the four aforementioned articles, selected 7 in total [6-12], and reported that probiotics were effective in preventing TD (sRR, 0.85; 95% CI, 0.79 to 0.91). In addition, a meta-analysis published in 2012 [13] included only 4 articles selected by Sazawal et al. [4] and demonstrated that their systemic review was incomplete.

Accordingly, what needs to be considered first and foremost for this discrepancy regarding the prevention efficacy of probiotics against TD between meta-analysis results and guidelines is that a systematic review should be adapted. The purpose of this study was to reevaluate the prevention efficacy of probiotics against TD using an adaptive meta-analysis.

## MATERIALS AND METHODS

Given that this study updated the existing meta-analysis, an adaptive meta-analysis was conducted [14]. This involved creating a list that synthesized articles by ‘cited by’ and ‘similar articles’ provided by PubMed (https://www.ncbi.nlm.nih.gov/pubmed/) for 2 meta-analysis studies [4,5] and their selected seven randomized trial articles [6-12].

The following are exclusion criteria applied to the synthesized list: (1) the subjects were not healthy adults, (2) the study design was not a randomized, placebo-controlled trial, (3) the treatment intervention was not a probiotic, and (4) the outcome was not the efficacy of TD prevention.

Data regarding the total number of subjects and the number of the subjects who developed TD in treatment and control groups were extracted from the finally selected articles. By applying the command *csi* in the Stata/SE version 14 (StataCorp., College Station, TX, USA), the relative risks (RRs) and 95% CIs were calculated for each article, after which a meta-analysis was conducted by calculating the sRR using the command *metan* [15]. Heterogeneity among the articles was assessed using the I-squared value (%), and this study applied a fixed effect model when there was no heterogeneity. To identify the existence of publication bias, this study applied the options *metafunnel* and *metabias*.

## RESULTS

**Figure 1.**
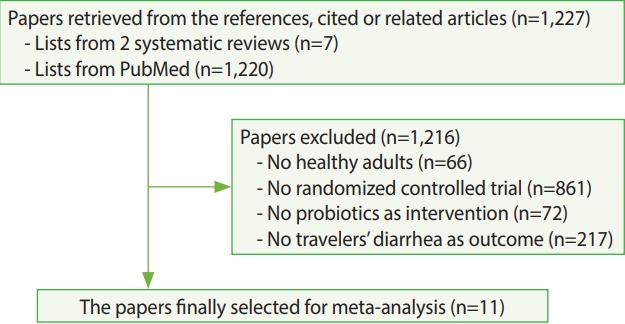
Flow chart of articles’ selection.

As of July 27, 2018, a list of 1,227 articles was created from the search, and 11 articles were finally selected when the four exclusion criteria were applied (Figure 1) [3,6-10,12,16-19]. Four more articles were selected in addition to the 7 articles chosen in the 2 existing meta-analysis studies. Furthermore, among the 7 articles selected in McFarland’s 2007 study [5], the article published by Kollaritsch et al. [11] was replaced by another article published by Kollaritsch & Wiedermann [19], which contained more detailed information. Table 1 shows the RRs and 95% CIs calculated based on information extracted from 11 articles.

**Figure 2.**
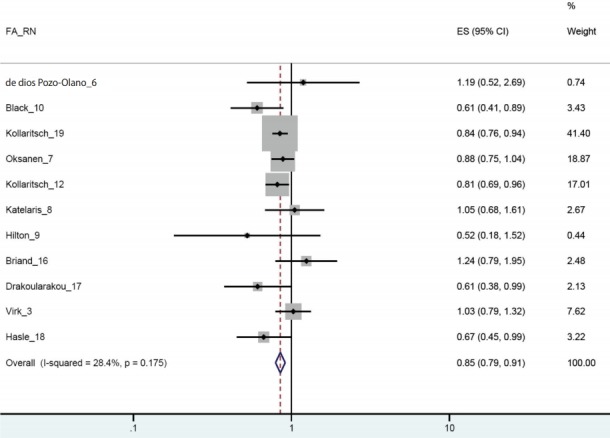
Forest plot from 11 selected articles. FA_RN, first author & reference number; ES, effect size.

**Figure 3.**
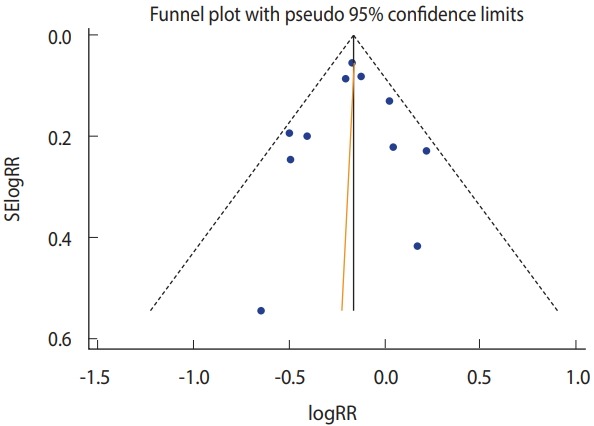
Funnel plot (Begg’s test: p=0.94; Egger’s test: p =0.83). SE, standard error; RR, relative risk.

Among the 11 selected articles, only two published since 2006 [16,18] presented intention-to-treat (ITT) and per-protocol (PP) separately. In this regard, when the RRs calculated by PP only were applied, there was efficacy in TD prevention while homogeneity was ensured (I-squared= 28.4%; sRR, 0.85; 95% CI, 0.79 to 0.91) (Figure 2). Statistical significance was ensured even when the value was replaced by ITT (sRR, 0.86; 95% CI, 0.80 to 0.92) (not shown). As a result of funnel plot, Begg’s test, and Egger’s test, it was confirmed that there was no publication bias (Figure 3).

## DISCUSSION

While the 2017 ISTM guideline [1] stated that there was insufficient evidence regarding the efficacy of probiotics in the prevention of TD, these results added evidence that probiotics had efficacy in preventing TD. Compared to McFarland’s effect size of 0.85 (95% CI, 0.79 to 0.91) from the seven selected articles [5], that of this study, which added 4 more articles, showed a similar level (sRR, 0.85; 95% CI, 0.79 to 0.91). Given that 2 out of the 4 added articles [3,16] produced the same effect size despite the lack of statistical significance in their RR, it provides evidence for the efficacy of probiotics in preventing TD.

As probiotics are already known to be effective for the management of acute infectious diarrhea and antibiotic-associated diarrhea [20], this meta-analysis in which they also showed prevention efficacy on TD would make it more necessary to conduct a follow-up study in the future. Nonetheless, considering that there were different types of probiotics that also differed in their routes of administration, the observation period varied among the articles, and the results were not classified according to PP and ITT, it would be necessary to ensure more consistency in performing clinical trials.

## Figures and Tables

**Table 1.** Summaries of 11 selected articles for adaptive meta-analysis

First author [RN]	Publication year	Treatment		Placebo		RR (95% CI)	Probiotics
		n	TD	n	TD		
de dios Pozo-Olano [6]	1978	26	9	24	7	1.19 (0.52, 2.69)	*L. acidophilus* & *L. bulgaricus*
Black [10]	1989	47	20	47	33	0.61 (0.41, 0.89)	Mixed
Kollaritsch [19]	1989	1,148	437	712	321	0.84 (0.76, 0.94)	*S. boulardii*
Oksanen [7]	1990	373	153	383	178	0.88 (0.75, 1.04)	*L. rhamnosus* GG
Kollaritsch [12]	1993	655	208	361	141	0.81 (0.69, 0.96)	*S. boulardii*
Katelaris [8]	1995	181	45	101	24	1.05 (0.68, 1.61)	*L. acidophilus* & *L. fermentum*
Hilton [9]	1997	126	5	119	9	0.52 (0.18, 1.52)	*L. rhamnosus* GG
Briand [16]	2006	79	30	72	22	1.24 (0.79, 1.95)	*L. acidophilus*
Drakoularakou [17]	2010	81	19	78	30	0.61 (0.38, 0.99)	GO
Virk [3]	2013	94	52	102	55	1.03 (0.79, 1.32)	Mixed
Hasle [18]	2017	167	32	167	48	0.67 (0.45, 0.99)	GO

RN, reference number; TD, travelers’ diarrhea; RR, relative risk; CI, confidence interval; *L, Lactobacillus*; *S, Saccharomyces*; GO, galacto-oligosaccharide.

## SUPPLEMENTARY MATERIALS

Supplementary Material: Korean version is available at (http://www.e-epih.org/).
