# Supplementary material for: Prophylactic efficacy of probiotics on travelers’ diarrhea: an adaptive meta-analysis of randomized controlled trials
Source: Epidemiol Health. 2018 Aug 29;40:e2018043. doi: 10.4178/epih.e2018043 (PMC6232657; doi:10.4178/epih.e2018043)
Supplement: Supplementary file 1 [file epih-40-e2018043-supplementary.pdf]

Volume: 40, Article ID: e2018043

https://doi.org/10.4178/epih.e2018043

<Brief Communication>

여행자설사에 있어 활생제의 예방효과: 무작위대조임상시험에 관한 개작 메타분석

Prophylactic Efficacy of Probiotics on Travelers' Diarrhea: An Adaptive Meta-analysis of Randomized Controlled Trials

Jong-Myon Bae

Department of Preventive Medicine, Jeju National University School of Medicine, Jejudo, Korea

Corresponding author: Jong-Myon Bae

jmbae@jejunu.ac.kr

064-755-5567

Volume: 40, Article ID: e2018043

https://doi.org/10.4178/epih.e2018043

Abstract

Objectives: The 2017 guideline for the prevention of travelers' diarrhea (TD) by International Society of Travel Medicine suggested that 'there is insufficient evidence to recommend the use of commercially available prebiotics or probiotics to prevent or treat TD'. However, a meta-analysis published in 2007 reported significant efficacy of probiotics in the prevention of TD (summary relative risk [sRR] =0.85, 95% confidence interval: 0.79-0.91). This study aimed to synthesize the efficacy of probiotics on TD by updating the meta-analysis of double-blind, placebo-controlled, randomized human trials.

Methods: The searching process was conducted by the adaptive meta-analysis method using 'cited by' and 'similar articles' options provided by PubMed. The inclusion criteria were double-blind, placebo-controlled, randomized human trials with hypotheses of probiotics as intervention and TD as outcome. The adaptive meta-analysis was conducted by Stata software using *csi, metan, metafunnel,* and *metabias* options.

Results: Eleven articles were selected for the meta-analysis. The sRR was 0.85 (95% confidence interval: 0.79-0.91) and showed statistical significance. There was no heterogeneity (I-squared=28.4%) and no publication bias.

Conclusion: Probiotics had statistically significant efficacy for the prevention of TD.

Keyword: Probiotics, Diarrhea, Randomized controlled trials, Meta-analysis

Volume: 40, Article ID: e2018043

https://doi.org/10.4178/epih.e2018043

Introduction

과거에 비하여 위생환경이 좋아졌다고 하지만, 전세계적인 인구 이동이 많아지면서 여행자 설사 (Travelers' diarrhea, TD)는 여전히 국제사회의 주요 보건문제이다 [1]. TD을 일으키는 병원균으로는 박테리아가 80% 이상을 차지한다는 점에서 [2], TD 발생을 사전에 예방하기 위한 항생제 투여를 고려할 수 있지만 항생제 내성의 문제가 야기된다 [3]. 국제여행자의학회 (International Society of Travel Medicine, ISTM)의 2017년 지침 [1]에 따르면, 일반인에 있어서 TD예방 목적의 항생제 투여를 금지하고 있다.

해당 지침은 또한 활생제 (probiotics)의 TD 예방효과에 대하여 근거가 부족하다고 밝히고 있다 (There is insufficient evidence to recommend the use of commercially available prebiotics or probiotics to prevent or treat travelers' diarrhea.). 이와 관련한 근거로 인용한 두 메타분석 논문 [4,5] 중 2006년도 발표된 Sazawal et al [4]은 무작위배정 위약대조군 임상시험결과를 발표한 논문 4편을 선정하였으며 [6-9], 예방효과에 있어서 통계적으로 유의한 차이가 없다고 발표하였다 (summary relative risk, sRR =0.92, 95% Confidence intervals, CI: 0.80-1.06). 그러나 1년 늦은 2007년에 발표한 McFarland [5]는 앞서의 4편에 3편을 추가한 7편을 선정한 결과 [6-12], 활생제가 TD 예방효과가 있다고 보고하였다 (sRR =0.85, 95%

CI: 0.79-0.91). 그리고 2012년에 발표된 메타분석 [13]은 Sazawal et al [4]이 선정한 4편만을 포함하여서, 체계적 고찰에 대한 불완전성을 확인하게 되었다.

따라서 활생제의 TD 예방효과에 대한 메타분석결과와 지침간의 이러한 괴리에 대하여 우선적으로 고려할 것은 체계적 고찰의 개작이 필요하다는 점이다. 본 연구의 목적은, 활생제의 TD 예방효과에 대하여 재검색을 통해 메타분석을 재수행하는 것이다.

Methods

기존의 메타분석을 개작한다는 점에서, adaptive meta-analysis를 적용하였다 [14]. 이는 2편의 메타분석 논문 [4,5]과, 선정된 총 7편의 무작위 임상시험 논문 [6-12] 각각에 있어 PubMed (https://www.ncbi.nlm.nih.gov/pubmed/)에서 제공하는 'cited by'와 'similar articles'의 문헌 목록을 수합하여 검색된 리스트를 만드는 것이다.

수합된 목록에서 선정을 위한 제외 기준들은 다음과 같다. (1) 대상자가 건강한 성인이 아닌 경우 (2) 연구설계가 무작위배정 위약대조 임상시험연구가 아닌 경우 (3) 처치개입이 활생제가 아닌 경우 (4) 성과가 TD 예방효과가 아닌 경우.

Volume: 40, Article ID: e2018043

https://doi.org/10.4178/epih.e2018043

최종 선정된 논문에 있어서 처치군과 비교군의 총 대상자 수와 TD 발생자수의 자료를 추출하였다. Stata SE 14 통계프로그램(www.stata.com)의 <csi> 명령어를 적용하여 논문 각각의 RR과 95% CI를 구하고, <metan> 명령어로 summary RR (sRR)을 산출하는 메타분석을 수행하였다 [15]. 논문들의 이질성은 I-squared value (%)로 평가하였고, 이질성이 없을 경우 fixed effect model을 적용하였다. 출판오류 (publication bias)를 알아보기 위하여 <metafunnel>과 <metabias> 옵션을 적용하였다

Results

2018년 7월 27일 현재, 검색을 통해 총 1,227편의 문헌 리스트가 만들어 졌으며, 4가지 제외기준을 적용하였을 때 최종 11편이 선정되었다 [3, 6-10,12,16-19] (Fig 1). 이는 기존의 두 메타분석 논문에서 선정한 7편 이외에 4편이 추가로 선정되었다. 또한 2007년에 발표한 McFarland [5]가 선정한 7편 중 Kollaritsch [11]은 보다 자세한 정보가 담긴 Kollaritsch [19]로 대치하였다. 11편에서 추출한 분석 정보에 따라 산출한 RR과 95% CI 은 Table 1과 같다.

11편 중 intention-to-treat (ITT)와 per-protocol(PP)을 구별해서 제시한 것은 2006년

이후 발표된 2편 [16,18]이었다. 이에 우선적으로 PP에 의해 구한 RR들을 적용하였을 때, 동질성을 확보한 가운데 (I-squared =28.4%) TD 보호효과가 있는 것으로 나왔다 (sRR =0.85; 95% CI: 0.79-0.91) (Fig 2). ITT 값으로 치환하였을 때도 통계적 유의성은 확보되었다 (sRR =0.86; 95% CI: 0.80-0.92) (not shown). 그리고 funnel plot, Begg't test, Egger's test 결과상 출판오류는 없는 것으로 확인되었다 (Fig 3).

Discussion

ISTM의 2017년 지침 [1]에서, 활생제의 TD 예방효과에 대하여 근거가 부족하다고 밝힌 가운데, 본 연구결과는 활생제는 TD 예방효과가 있다는 근거를 추가하게 되었다. 7편에 대한 McFarland [5]의 효과크기가 0.85 (95% CI: 0.79-0.91)에 비교하여, 4편을 추가한 본 연구의 효과크기는 동일 수준을 보였다 (sRR =0.85; 95% CI: 0.79-0.91). 추가된 4편 중 두 편 [3, 16]에서 RR의 통계적 유의성이 없음에도 불구하고 같은 효과크기가 산출되었다는 점에서, 활생제의 TD 예방효과에 대한 근거를 지지한다.

활생제는 급성감염설사 (acute infectious diarrhea), 항생제투여로 인한 설사 (antibiotic-associated diarrhea) 등에 효과적이라고 이미 알려져 있어 [20], 이번 메타분석으

Volume: 40, Article ID: e2018043

https://doi.org/10.4178/epih.e2018043

로 TD에 대하여도 활생제가 예방효과가 있다는 점은 향후 추후 연구의 필요성이 대두된다. 그러나 활생제의 종류와 투여법이 다양하고, 관찰기간 등이 연구마다 다르며, 결과제시에서 PP와 ITT를 구별하지 않다는 점 등에서 임상시험 연구수행의 통일성을 기할 필요가 있겠다.

.

Volume: 40, Article ID: e2018043

https://doi.org/10.4178/epih.e2018043

References

1. Riddle MS, Connor BA, Beeching NJ, DuPont HL, Hamer DH, Kozarsky P, et al. Guidelines for the prevention and treatment of travelers' diarrhea: a graded expert panel report. J Travel Med. 2017;24(suppl_1):S57-S74.

2. Gascón J. Epidemiology, etiology and pathophysiology of traveler's diarrhea. Digestion. 2006;73 Suppl 1:102-8.

3. Virk A, Mandrekar J, Berbari EF, Boyce TG, Fischer PR, Kasten MJ, et al. A randomized, double blind, placebo-controlled trial of an oral synbiotic (AKSB) for prevention of travelers' diarrhea. J Travel Med. 2013;20(2):88-94.

4. Sazawal S, Hiremath G, Dhingra U, Malik P, Deb S, Black RE. Efficacy of probiotics in prevention of acute diarrhoea: a meta-analysis of masked, randomised, placebo-controlled trials. Lancet Infect Dis. 2006;6(6):374-82.

5. McFarland LV. Meta-analysis of probiotics for the prevention of traveler's diarrhea. Travel Med Infect Dis. 2007;5(2):97-105.

6. de dios Pozo-Olano J, Warram JH Jr, Gómez RG, Cavazos MG. Effect of a lactobacilli preparation on traveler's diarrhea. A randomized, double blind clinical trial. Gastroenterology. 1978;74(5 Pt 1):829-30.

7. Oksanen PJ, Salminen S, Saxelin M, Hämäläinen P, Ihantola-Vormisto A, Muurasniemi-Isoviita L, et al. Prevention of travellers' diarrhoea by Lactobacillus GG. Ann Med. 1990;22(1):53-6.

8. Katelaris PH, Salam I, Farthing MJ. Lactobacilli to prevent traveler's diarrhea? N Engl J Med. 1995;333(20):1360-1.

9. Hilton E, Kolakowski P, Singer C, Smith M. Efficacy of Lactobacillus GG as a Diarrheal Preventive in Travelers. J Travel Med. 1997;4(1):41-43.

10. Black FT, Anderson PL, Orskov J, Orskov F, Gaarslev K. Laulund S. Prophylactic efficacy of Lactobacilli on traveler's diarrhea. Travel Med. 1989;7:333-335.

11. Kollaritsch H, Kremsner P, Wiedermann G, Scheiner O. Prevention of traveller's diarrhea: comparison of different nonantibiotic preparations. Travel Med Int. 1989:6:9–17.

12. Kollaritsch H, Holst H, Grobara P, Wiedermann G. Prophylaxe der reisediarrhoe mit Saccharomyces boulardii [Prevention of traveler's diarrhea with Saccharomyces boulardii. Results of a placebo controlled double-blind study]. Fortschr Med. 1993;111:152–6.

13. Ritchie ML, Romanuk TN. A meta-analysis of probiotic efficacy for gastrointestinal diseases. PLoS One. 2012;7(4):e34938.

14. Bae JM, Kim EH. Citation discovery tools for conducting adaptive meta-analyses to update systematic reviews. J Prev Med Public Health. 2016;49:129-133.

15. Shim SR, Shin IS, Bae JM. Intervention meta-analysis using STATA software. J Health Info Stat. 2016;41(1):123-134. (korean)

16. Briand V, Buffet P, Genty S, Lacombe K, Godineau N, Salomon J, et al. Absence of efficacy of nonviable Lactobacillus acidophilus for the prevention of traveler's diarrhea: a randomized, double-blind, controlled study. Clin Infect Dis. 2006;43(9):1170-5.

17. Drakoularakou A, Tzortzis G, Rastall RA, Gibson GR. A double-blind, placebo-controlled, randomized human study assessing the capacity of a novel galacto-oligosaccharide mixture in reducing travellers' diarrhoea. Eur J Clin Nutr. 2010;64(2):146-52.

18. Hasle G, Raastad R, Bjune G, Jenum PA, Heier L. Can a galacto-oligosaccharide reduce the

risk of traveller's diarrhoea? A placebo-controlled, randomized, double-blind study. J Travel Med. 2017;24(5).

19. Kollaritsch HH, Wiedermann G. Prevention of traveler's diarrhea: a double-blind randomized trial with Saccharomyces cerevisiae Hansen CBS 5926. Travel Med. 1989:7;328-332.

20. Wilkins T, Sequoia J. Probiotics for Gastrointestinal Conditions: A Summary of the Evidence. Am Fam Physician. 2017;96(3):170-178.

Volume: 40, Article ID: e2018043

https://doi.org/10.4178/epih.e2018043

Table 1. Summaries of 11 selected articles for adaptive meta-analysis*

Reference number	First Author	Publication Year	Treatment group		Placebo group		RR [95% CI]	Probiotics
			N	TD	N	TD		
6	Pozo-Olano	1978	26	9	24	7	1.19 [0.52, 2.69]	*L. acidophilus & L. bulgaricus*
10	Black	1989	47	20	47	33	0.61 [0.41, 0.89]	mixed
19	Kollaritsch	1989	1148	437	712	321	0.84 [0.76, 0.94]	*S. boulardii*
7	Oksanen	1990	373	153	383	178	0.88 [0.75, 1.04]	*L. rhamnosus* GG
12	Kollaritsch	1993	655	208	361	141	0.81 [0.69, 0.96]	*S. boulardii*
8	Katelaris	1995	181	45	101	24	1.05 [0.68, 1.61]	*L. acidophilus & L. fermentum*
9	Hilton	1997	126	5	119	9	0.52 [0.18, 1.52]	*L. rhamnosus* GG
16	Briand	2006	79	30	72	22	1.24 [0.79, 1.95]	*L. acidophilus*
17	Drakoularakou	2010	81	19	78	30	0.61 [0.38, 0.99]	GO
3	Virk	2013	94	52	102	55	1.03 [0.79, 1.32]	mixed
18	Hasle	2017	167	32	167	48	0.67 [0.45, 0.99]	GO

Volume: 40, Article ID: e2018043

https://doi.org/10.4178/epih.e2018043

* CI: confidence interval; GO: galacto-ologisaccharide; L.: Lactobacillus; RR: relative risk; S.: Saccaromyces; TD : travelers' diarrhea
